# Idiopathic pulmonary fibrosis: prognostic impact of histologic honeycombing in transbronchial lung cryobiopsy

**DOI:** 10.1186/s40248-019-0170-y

**Published:** 2019-02-08

**Authors:** Claudia Ravaglia, Marcello Bosi, Athol U. Wells, Carlo Gurioli, Christian Gurioli, Alessandra Dubini, Sara Piciucchi, Silvia Puglisi, Susanna Mascetti, Antonella Arcadu, Sara Tomassetti, Venerino Poletti

**Affiliations:** 1 Department of Thoracic Diseases, G.B. Morgagni - L. Pierantoni Hospital, Via C. Forlanini 34, 47121 Forlì, FC Italy; 2 Interstitial Lung Disease Unit, Royal Brompton Hospital, London, UK; 3 Department of Pathology, G.B. Morgagni - L. Pierantoni Hospital, Forlì, Italy; 4 Department of Radiology, G.B. Morgagni - L. Pierantoni Hospital, Forlì, Italy; 5 Department of Respiratory Diseases and Allergy, Aarhus University Hospital, Aarhus, Denmark

**Keywords:** Transbronchial lung cryobiopsy, Honeycombing, Lung biopsy, Cryobiopsy, Interstitial lung disease

## Abstract

**Background:**

Prognostic evaluation in idiopathic pulmonary fibrosis (IPF) may be important as it can guide management decisions, but the potential role of honeycomb changes in providing information about outcome and survival of patients with IPF, particularly if diagnosed using cryobiopsy, has not been evaluated. Aim of this study was to determinate whether a relationship exists between honeycombing on cryobiopsy and clinical/radiological picture and outcome in patients with IPF and to assess whether the same pathologic criteria that have been used to define the UIP pattern (usual interstitial pneumonia) for surgical biopsy can also be applied to cryobiopsy.

**Methods:**

Sixty-three subjects with a multidisciplinary diagnosis of IPF and a UIP pattern on cryobiopsy were evaluated. Patients were classified into two sub-groups depending on the presence of honeycombing on histology.

**Results:**

The presence of honeycombing on cryobiopsy did not identify a specific phenotype of patients as it did not correlate with radiological and clinical picture and it was not associated neither with the risk of death (*p* = 0.1192) or with the event-free survival (*p* = 0.827); a higher number of samples and the presence of pleura on biopsy were instead associated with an increase in the finding of honeycombing.

**Conclusions:**

The same pathologic criteria that have been used to define the UIP pattern in surgical biopsies (with honeycombing changes considered as non-mandatory for the definition of the pattern itself) can be applied to cryobiopsy samples, as the presence of these changes do not define different clinical or radiological phenotypes of patients with IPF.

## Background

Prognostic evaluation in Idiopathic Pulmonary Fibrosis (IPF) may be important as it can guide management decisions. Correlation between lung pathologic features and survival has been controversial in the past; a significant number of more recent studies have highlighted the role of fibroblastic foci (FF) as predictor of physiologic decline and mortality. However, in the literature there are no papers showing the potential role of honeycomb changes in providing information about outcome and survival of patients with IPF, particularly in patients diagnosed using transbronchial lung cryobiopsy (TLCB). The aim of this study was to determinate whether a relationship exists between pathologic honeycombing and clinical picture, radiological features and mortality in patients with a multidisciplinary diagnosis of IPF obtained with transbronchial lung cryobiopsy.

## Methods

This study was submitted by the Area Vasta Romagna Ethical Committee. Prior to the procedure, written informed consent has been obtained from all subjects. Sixty-three subjects who had undergone transbronchial lung cryobiopsy (TLCB) at the Pulmonology Unit of G.B. Morgagni– L. Pierantoni Hospital in Forlì (Italy) for suspected diffuse parenchymal lung disease from January 2013 to December 2016 were retrospectively identified from the Interstitial Lung Disease (ILD) database.

All subjects had clinical information, serology testing and high-resolution computed tomography (HRCT) scans compatible with a fibrotic ILD, but insufficient elements to achieve an IPF diagnosis by current international consensus criteria; for all included patients, final multidisciplinary diagnosis was IPF after achieving a morphological picture compatible with UIP pattern on cryobiopsy. HRCT images were made within 1 month prior to the bronchoscopy and CT scans were analyzed by a dedicated radiologist (SP) under standard clinical care.

Bronchoscopies were performed as previously described [1, 2] under fluoroscopic guidance: 1.9 mm/2.4 mm cryoprobes were used (ERBE, Germany), patients were deeply sedated with intravenous propofol and remifentanil and intubated with a rigid tracheoscope, maintaining spontaneous breathing. Bronchoscopic cryobiopsy was targeted to the areas of abnormality seen on HRCT, therefore samples have been taken from either one site or multiple sites depending on the radiological pattern. When more than one site was sampled, UIP pattern had to be observed in at least one of the sampled sites. Once brought into position, the probe was cooled for approximately 5–6 s in case of 2.4 mm probe or 7–8 s in case of 1.9 mm probe, then it was retracted with the frozen lung tissue being attached on the probe’s tip. The frozen specimen was thawed in saline and then transferred to formalin for fixation. To prevent severe bleeding, we used Fogarty balloon in each procedure, placing it in the lobar bronchus near the biopsy segment and inflating it always after biopsy, then immediately deflating it in case of absence of hemorrhage or after bleeding cessation.

**Fig. 1:**
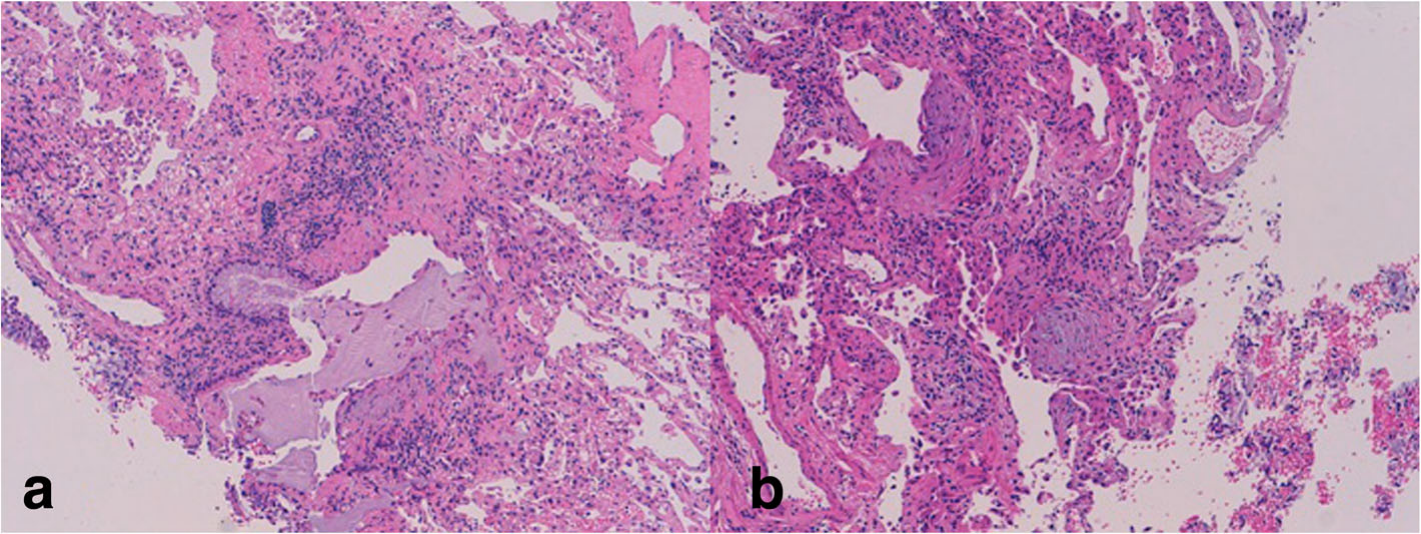
Transbronchial lung cryobiopsy showing UIP pattern resulting from a combination of patchy fibrosis, fibroblastic foci and honeycombing (**1a**) and from a combination of patchy fibrosis and fibroblastic foci only without honeycombing (**1b**)

Biopsy specimens were fixed in formalin and embedded in paraffin. Hematoxylin and eosin (H&E) stained slides were reviewed by the standard protocol of the Morgagni Hospital clinical pathology laboratory. Specimens were reviewed under standard clinical care by two expert lung pathologists (AD and VP). The pathologic diagnosis of usual interstitial pneumonia (UIP) was made according to the criteria listed in the current guidelines [3–5]: for the identification of a UIP pattern, the morphologic descriptors were dense fibrosis with architectural distortion, predominant sub-pleural and/or para-septal distribution of fibrosis, fibroblastic foci, patchy involvement of lung parenchyma by fibrosis and absence of features suggesting alternative diagnoses; moreover, patients were classified into two sub-groups depending on the presence of honeycombing on histology: group A included patients with a combination of patchy fibrosis, fibroblastic foci and honeycombing; group B included patients with patchy fibrosis and fibroblastic foci, without honeycombing (Fig. 1). The exclusion criteria were: (a) known cause of pulmonary fibrosis (connective tissue diseases, occupational and/or environmental exposure, drugs, etc.); (b) no histology available; (c) pathologic findings consistent with NSIP (non-specific interstitial pneumonia), DIP (desquamative interstitial pneumonia), RB-ILD (respiratory bronchiolitis-interstitial lung disease), LIP (lymphocytic interstitial pneumonia), DAD (diffuse alveolar damage), eosinophilic granuloma, HP (hypersensitivity pneumonitis), sarcoidosis, OP (organizing pneumonia); (d) ancillary findings on histology diagnostic of secondary UIP (isolated giant cells and/or tiny granulomas in the inter-alveolar interstitium, chronic inflammatory infiltrate of the pleura and interstitium, bridging fibrosis, asbestos bodies, bronchiolocentric damage, lymphoid follicles with germinal centers, alveolar and interstitial eosinophils, IgG4 positive plasma cell infiltrate, alveolar damage with/without organizing pneumonia). Clinical information, radiological features and biopsy results were then reviewed by clinicians, radiologist and pathologists in a multidisciplinary discussion and only patients with a final diagnosis of IPF were included in the study.

### Statistical analysis

Data are presented using descriptive statistics (median value, min-max). The differences between the groups were analyzed using the Mann Whitney test or the Kruskal Wallis variance test, while the *post-hoc* analysis was performed, when necessary, with the Dunn multiple comparison test. Comparisons between percentages were performed with Chi-square test and Fisher’s exact test. Survival curves were estimated using the Kaplan-Meier method and compared with the log-rank test (Mantel-Cox). Patients were considered censored if they 1) were still alive when last contacted (censored at the last status date), 2) had received a lung transplant (censored at the time of the transplant). Survival time was calculated as the time since lung biopsy. Logistic regression analysis was used to identify independent variables as predictors of honeycombing. The level of statistical significance was assumed for *p* < 0.05. All statistical analyzes were conducted using IBM SPSS version 19.

## Results

During the study period, 63 subjects undergoing TLCB were included. Honeycombing was described on histology in 18 patients (group A, 28.6%) while in 45 patients (group B, 71.4%) honeycombing was not identified. Subject characteristics are summarized in Table 1: median age of patients at diagnosis was 64 years (range 45–78) and male were 71%; median baseline forced vital capacity (FVC) was 86.0% (range 59–136), median baseline diffusing capacity of the lungs for carbon monoxide (DL_CO_) was 56.0% (range 23–117).

**Table 1:** Clinical and radiological features of patients submitted to trans-bronchial lung criobiopsy (TLCB)

	All patients (N 63)	Group A = with HC (N 18)	Group B = no HC (N 45)	*p*
Age, Y	64 (45–78)	65 (53–76)	63 (45–78)	0,081 (^a^)
Gender M/F (N)	45/18	11/7	34/11	0,354 (^b^)
HRCT pattern: probable UIP/ indeterminate for UIP (N)	39/24	13/5	26/19	0,329 (^b^)
FVC, % predicted	86,0 (59–136)	85,5 (59–110)	86,0 (59–136)	0,341 (^a^)
DL_CO_, % predicted	56 (23–117)	48 (35–117)	58 (23–99)	0,089 (^a^)
Samples, N	3 (1–5)	3 (1–5)	4 (1–5)	0,373 (^a^)
Site of samples: 1 lobe/2 lobes (N)	11/52	3/15	8/45	1000 (^b^)
Pleura (Y/N)	13/50	4/14	9/36	1000 (^b^)
Death, N (%)	10 (16%)	4 (22%)	6 (13%)	0,452 (^b^)

*HC* honeycombing, (^a^) = Mann-Whitney test, (^b^) = Chi-square test, *FVC* forced vital capacity, *DLCO* diffusing capacity of the lungs for carbon monoxide

**Fig. 2:**
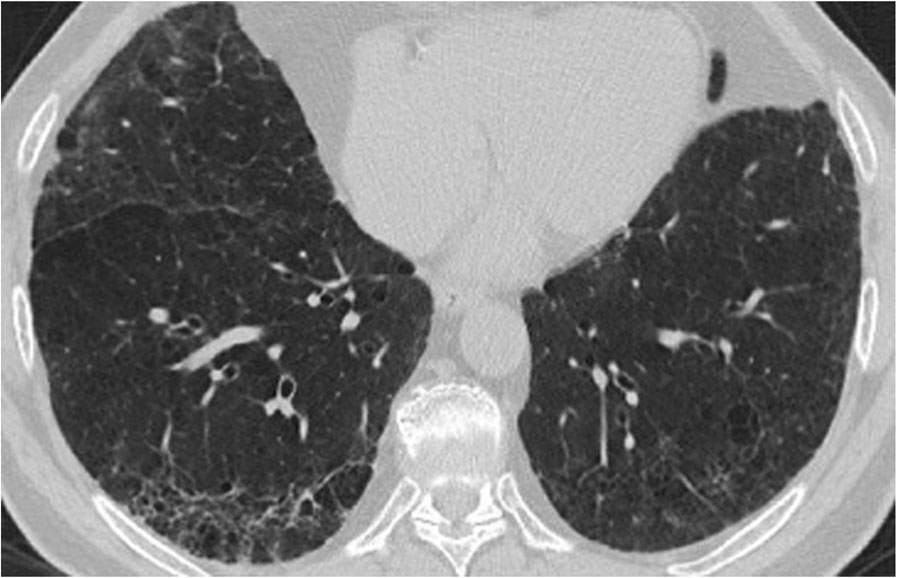
Chest HRCT showing a reticular pattern with peripheral traction bronchiectasis, with a predominant basal and sub-pleural distribution (probable UIP pattern)

**Fig. 3:**
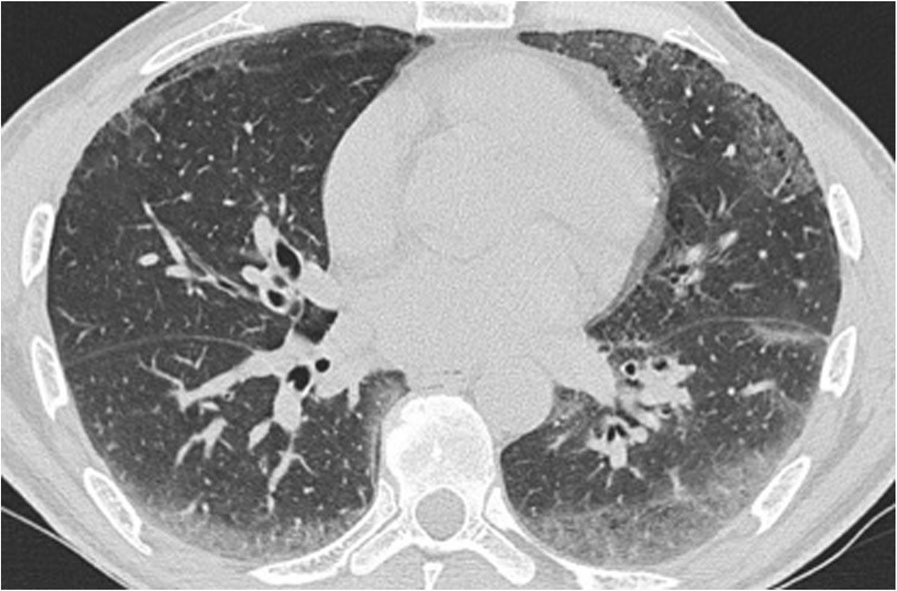
Chest HRCT showing a CT pattern indeterminate for UIP (fibrosis with ground-glass suggestive of non-UIP pattern)

Age at diagnosis, gender distribution and lung function tests did not differ between the two pathologic groups (age: *p* = 0.081, Mann-Whitney test; gender: *p* = 0.354, Fisher’s exact test respectively; FVC: p = 0,341, Mann-Whitney test; DL_CO_: p = 0,089, Mann-Whitney test) (Table 1). Thirty-nine patients (61,9%) had a CT characterized by a reticular pattern with peripheral traction bronchiectasis or bronchiolectasis, no honeycombing and absence of radiological features suggesting an alternative diagnosis, with a predominant basal and sub-pleural distribution (pattern that is now called “probable UIP” according to the most recent guidelines) [3–6] (Fig. 2). In 24 patients (38,1%), the CT showed evidence of fibrosis with variable or diffuse distribution and some inconspicuous features suggestive of non-UIP pattern (which corresponds to the new term “indeterminate” from the new guidelines) [3–6] (Fig. 3). The same distribution of radiological pattern was found in the two pathologic groups (*p* = 0,329, Chi-square test).

In 11 patients (17,5%) biopsies were taken from one lobe and in 52 patients (82,5%) from two different lobes; there was no statistical difference in terms of sampling strategy between the group in which honeycombing was identified and the group with no honeycombing (*p* = 1,000). The choice of the site and side of biopsy was guided by HRCT and was decided before the procedure. Average number of fragments was 3 (range 1–5), with no differences between the two groups in terms of samples number (*p* = 0,373, Mann-Whitney test). Pleural tissue was detected in 13 cases (20,6%) (Table 1).

Logistic regression analysis was performed using the presence of honeycombing on cryobiopsy as the dependent variable; independent variables were clinical features (age at diagnosis, gender, FVC, DL_CO_), radiological pattern, number of samples, number of sites of sampling and presence of pleura. The number of samples and the presence of pleura were independently correlated with pathologic honeycombing: every sample increases the honeycombing probability of 136% and the absence of pleura decreases the probability of 93% (Nagelkerke R-square 0.438; number of samples: B 0.860, p 0.037, Exp-B 2.363; absence of pleura: B - 2.646 p 0.001, Exp-B 0.071).

Forty-seven subjects had received treatment for their illness during the study period: 43 patients had been treated with anti-fibrotic drugs (31 pirfenidone, 12 nintedanib), corticosteroids were administered in 20 patients, NAC (N-acetyl cysteine) in 12 patients, immunosuppressive drugs in 5 patients (4 azathioprine, 1 mofetil mycophenolate) and 16 subjects had never received treatment directed at IPF.

**Fig. 4:**
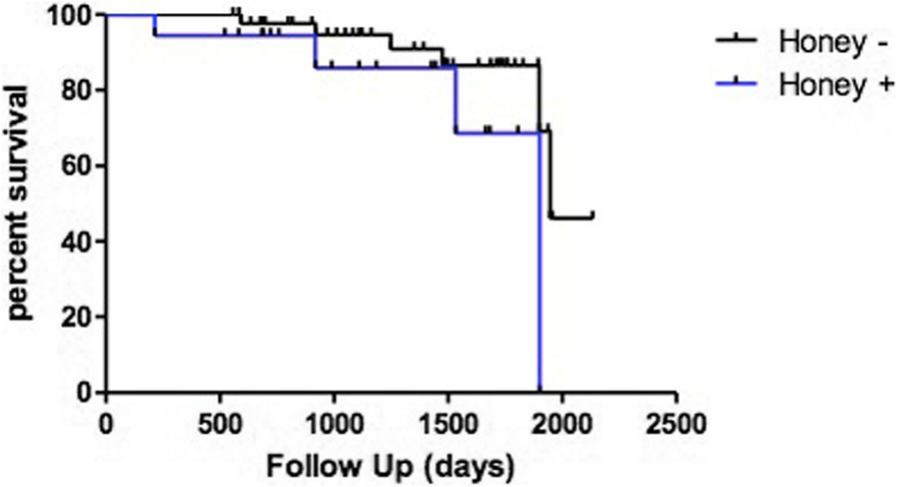
Kaplan-Meier curve of patients enrolled in the study. There was no prognostic difference between the groups of patients (with or without honeycombing on histology) (log-rank [Mantel-Cox] test: chi square 2.428, *p* = 0.1192)

**Fig. 5:**
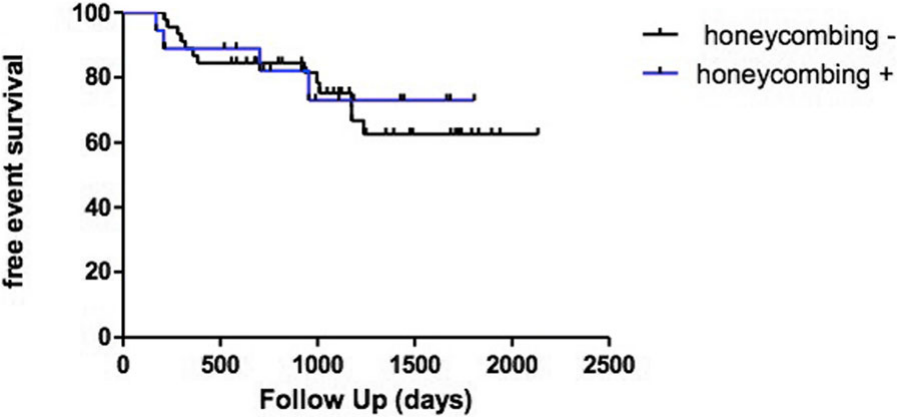
Kaplan-Meier curve of TLCB patients. There was no correlation between free-event survival and presence of honeycombing on histology (log-rank Mantel-Cox test chi square 0.047, *p* = 0.827)

The median length of follow up from the time of the biopsy was 41 months; 10 patients (15.8%) died during the follow up period and none of the deaths occurred within 30 days of the procedure or was related to the procedure itself. Death and survival rate were not related with the presence of honeycombing (Table 1) (in the analysis, we censored those patients who underwent lung transplantation). We reviewed the prognosis and clinical course for all patients enrolled in this study to draw the Kaplan-Meier curve. Significant prognostic differences between the groups (with or without honeycombing on histology) were not demonstrated using log-rank Mantel-Cox test (chi square 2.428, p 0.1192, Fig. 4) and the presence of honeycombing was not significantly associated with an increased risk of death. Finally, the presence of honeycombing on histology did not influence the event-free survival, with events being 1) progression of the disease, 2) acute exacerbation and 3) death) (*p* = 0.827, log-rank Mantel-Cox test, Fig. 5).

## Discussion

Accurate prognostic evaluation in IPF could be important as it can guide management decisions. Over the past 20 years, several papers have reported clinical, radiological and pathologic features which may influence the outcome in ILDs. The pathologic features of UIP are characterized by a temporally and spatially heterogeneous intermix of normal lung tissue, acellular bundles of collagen, microscopic honeycombing and fibroblastic foci (myxoid matrix containing aggregates of actively proliferating and collagen-producing myofibroblasts). The correlation between lung pathology and survival has been controversial in the past [7]. Old studies of outcome and histopathology had shown that prognosis of IIPs can be determined by the extent of fibrosis and cellularity on biopsy [8–10]; other studies have found that the degree of alveolar space granulation tissue deposition and the extent of young connective tissue within the fibroblastic foci were the only pathologic features predictive of survival in IPF [11]. A significant number of more recent studies have highlighted the very role of fibroblastic foci as predictor of physiologic decline and mortality [12–14].

In IPF, most fibroblast foci are observed within honeycomb cysts; however, the presence of honeycombing is not always required for the diagnosis of UIP pattern [4, 5]. Areas of honeycomb change are composed of cystic fibrotic airspaces, which are frequently lined by bronchiolar epithelium and filled with mucin [3–5, 15]. In reality, morphologic analysis of honeycombed areas in IPF reveals prominent bronchiolar changes which sometimes can mimic epithelial neoplasms; this abnormal proliferation of bronchiolar structures seems to be the result of deranged proliferation of distal airways that colonize the alveolar parenchyma progressively [16]. There are no papers in the literature showing the potential role of honeycomb changes, observed in surgical lung biopsies, in providing information about outcome and survival of patients with IPF. Furthermore, to date no attempts have been made to determine whether a relationship exists between pathologic honeycombing observed in transbronchial lung cryobiopsy and clinical picture, radiological features or mortality.

The key observation in our study is that honeycombing on pathologic analysis of cryobiopsy does not correlate with outcome; as a matter of fact, the presence of honeycombing was not significantly associated with an increased risk of death and with the event-free survival (evaluated including the progression of the disease, acute exacerbations and death). These results give further support to what is reported in the guidelines: honeycombing changes are not mandatory to define the UIP pattern on biopsy samples; in fact, pathologic honeycombing does not seem to define different clinical and radiological profiles of patients with IPF. Until now, UIP pattern has been defined in the literature and in the current clinical practice through criteria and morphological features deriving only from surgical lung biopsy. Recent papers about diagnostic yield of cryobiopsy in fibrotic ILDs have directly applied these criteria to the samples obtained by cryoprobe [1, 17–21]; the impact of TLCB on the multidisciplinary process in patients with atypical radiological picture had been similar to that of surgical lung biopsy (SLB) [22]. These data have already supported the role of TLCB in the evaluation of patients with suspected IPF and have activated a series of efforts aimed at standardizing the procedure [23]; however, until now the prognostic significance of morphological features had not been evaluated in samples obtained by cryobiopsy.

Another important point in our study is that similarly to what happens with surgical lung biopsies, pathologic honeycombing does not even correlate with radiological pattern and clinical picture and does not seem to identify a specific clinical or radiological phenotype: for example, it is not more frequently observed in older patients with more compromised lung function or it is not more frequently described in patients with a probable UIP pattern rather than in patients with an indeterminate pattern on CT [5].

Honeycombing was not influenced by the sampling strategy: it was not more frequently observed when biopsies were taken from more than one site. This could be explained by the fact that cryobiopsy was performed in different lobes in patients with significant radiographic inter-lobar heterogeneity, while it was more frequently performed in different segments of the same lobe in patients with diffuse radiographic pattern (in the upper and the lower lobes) or in patients with a significant apical-basal gradient. However, the number of samples and the presence of pleura on biopsy were independently correlated with pathologic honeycombing. Usually 2–6 biopsies [24, 25] are taken in the common clinical practice: multiple biopsies are undertaken to reduce sampling error, even if the optimal protocol has not been standardized yet. Two samples instead of only one can increase the diagnostic yield of cryobiopsy in diffuse parenchymal lung diseases [25] and it is quite simple to accept that the number of biopsies can also contribute to a significant increase in the finding of honeycombing. Regarding the presence of pleura, ideally TLCB samples the middle third of the lung tissue between the large airways and the pleura, about 1 cm from the pleura itself [26]; however, more peripheral lung tissue, including visceral pleura, is not infrequently encountered. In previous studies, approximately 30% of the samples obtained by cryobiopsy show the presence of pleura [2, 22]. On the other hand, UIP changes are usually localized in the sub-pleural regions of the lower lobes in IPF [3–6, 27] and it is not surprising that the presence of pleura in the tissue is correlated with honeycombing itself.

Our study has some limitations. First, it was retrospective in design. However, all diagnoses have been made based on current accepted radiological and pathologic criteria and multidisciplinary discussion [3, 27]; the survival Kaplan-Meier curves have been drawled considering the presence of honeycombing on histology. We know from the literature that TLCB appears to be safer than surgical lung biopsy; our previous meta-analysis has revealed an overall mortality rate with this procedure of about 0.1% among approximately 1,000 patients [2]. The analysis of more recent data published in the literature on cryobiopsy documents 7 deaths within a month after the procedure [1, 28–30]. In the present study, we did not observe deaths related to acute exacerbation following the sampling procedure. The risk of acute exacerbation needs to be assessed before the procedure, particularly in case of recent worsening [31–34]: our data demonstrate that TLCB can be applicable in older patients and/or patients with more severe functional deterioration compared to patients feasible for surgery. A second limitation of our study could be that we have not described in detail the effect of pharmacological treatments on the observed results; however, the 2 groups (A and B) were homogeneous from the point of view of medication and these did not influence outcome and mortality. Another possible limitation of the study is that we included only patients with lung biopsy, therefore patients with imaging and clinical parameters insufficient for a high confidence diagnosis of IPF; our patient cohort may not replicate general population with IPF, in which a significant portion of patients do not need lung biopsy. Finally, the cellularity factor (severity and extent of cellular infiltration of the alveolar walls), fibroblastic foci profusion and inter-observer agreement between the two pathologists were not evaluated.

## Conclusions

Despite the above limitations, this simple retrospective study can give very important information, especially as it concerns aspects that have never been taken into consideration in the several papers about transbronchial lung cryobiopsy. The identification of honeycomb areas on transbronchial lung cryobiopsy has no prognostic implications and do not define the clinical and radiological profiles of patients. Finally, we can state that the same pathologic criteria that have been used to define the UIP pattern for surgical lung biopsy samples can also be applied to the lung tissue samples obtained by cryobiopsy, with honeycombing changes considered as non-mandatory for the definition of pattern itself.

## Abbreviations

DAD: diffuse alveolar damage
DIP: desquamative interstitial pneumonia
DL_CO_: diffusing capacity of the lungs for carbon monoxide
FF: fibroblastic foci
FVC: forced vital capacity
H&E: hematoxylin and eosin
HP: hypersensitivity pneumonitis
HRCT: high-resolution computed tomography
ILD: Interstitial Lung Disease
IPF: idiopathic pulmonary fibrosis
LIP: lymphocytic interstitial pneumonia
NAC: N-acetyl cysteine
NSIP: non-specific interstitial pneumonia
OP: organizing pneumonia
RB-ILD: respiratory bronchiolitis-interstitial lung disease
TLCB: transbronchial lung cryobiopsy
UIP: usual interstitial pneumonia
